# Millstone Exfoliation: a True Shear Exfoliation for Large-Size Few-Layer Graphene Oxide

**DOI:** 10.1186/s11671-018-2598-y

**Published:** 2018-06-20

**Authors:** Heng-Ju Yoon, Jae Young Lee, Tae-Ho Yoon

**Affiliations:** School of Materials Science and Engineering, Gwangju Institute of Science and Engineering (GIST), 123 Cheomdangwagi-ro, Buk-gu, Gwangju, 61005 South Korea

**Keywords:** Graphene oxide, Millstone exfoliation, Mild oxidation

## Abstract

A millstone (MS) was introduced in the production of large-size few-layer-graphene oxide (FLGO) via true shear exfoliation in order to minimize fragmentation. The MS was constructed with two glass plates, where the top plate was designed to rotate against the stationary bottom plate, thereby generating true shear force. Mildly oxidized graphite (MOG) was used for MS exfoliation in order to obtain both good property and high yield. The rpm of rotation (10, 20, 30, 40, and 50), solution concentration (0.5, 1, and 2 mg/ml), and number of exfoliation (1, 2, and 3) were optimized by measuring the UV-vis absorption, and the effect of oxidation time (30, 60, and 90 min) was studied under the given optimum conditions. Next, the FLGO was isolated by centrifugation and characterized by TEM and AFM. The FLGO obtained was as large as ~ 10 μm in size, which was slightly smaller than the pristine graphite, suggesting a possibility of slight fragmentation. But it was still much larger than the FLGO obtained via sonication (< 1 μm), demonstrating successful MS exfoliation.

**Electronic supplementary material:**

The online version of this article (10.1186/s11671-018-2598-y) contains supplementary material, which is available to authorized users.

## Background

Excellent properties [1] of graphene have led to extensive studies on their applications in field-effect transistors [2, 3], sensors [4, 5], transparent electrodes [6, 7], and other areas [8–10]. For such applications, it is essential to have graphene with high quality and affordable price [11], as well as large size, to minimize the inter-particle connectivity problem [12]. Consequently, various methods have been introduced to prepare graphene, such as chemical vapor deposition (CVD) [13] and epitaxial growth [14]. These methods generated high-quality graphene with large size but had high costs. The exfoliation of inexpensive natural graphite, on the other hand, resulted in graphene at low cost, but the size of graphene was limited by the size of pristine graphite, with even smaller-size graphene being reported at times due to fragmentation upon exfoliation.

Physical exfoliation, such as sonication [15, 16], ball milling [17, 18], and shear exfoliation [19, 20], produced high-quality graphene, but these methods generally produced graphene with small size and low yield [21]. In comparison, chemical exfoliation generally produced larger-size graphene oxide (GO) with higher yield [22] than the physical exfoliation, but the GO produced was smaller in size than the pristine graphite, in general. This was attributed to fragmentation arising from the additional exfoliation such as sonication, which was carried out after oxidation to increase the yield of GO or large-size few-layer-graphene oxide (FLGO) [23]. Additionally, the oxidation-fragmentation occurring from harsh oxidation conditions [24, 25] could have played a contributing role.

Thus, in order to avoid such fragmentation and to produce large-size GO, two possible approaches can be considered. One is the optimization of oxidation conditions to afford complete oxidation-exfoliation with minimum oxidation-fragmentation and the other is the modification of the existing exfoliation methods or the introduction of a new method to afford complete exfoliation with no or minimum fragmentation. Moreover, it would be necessary to employ large-size graphite since the GO size is limited by the size of the pristine graphite. In fact, studies on millimeter- to few-hundred-micron-size graphite [26–31] reported much larger GO than the ones obtained from the widely used 325-mesh graphite [22].

Concerning the first approach, three types of oxidation conditions were studied in the literature: (1) two-step oxidation [26–29]; (2) preparation of graphite intercalation compound (GIC) or expanded graphite, followed by exfoliation [32–34]; and (3) oxidation under harsher conditions than the ones used in the Hummers method [35–37]. These methods produced much larger GO than the previously reported methods, but the GO size was still smaller than the pristine graphite, indicating that oxidation-fragmentation occurred [24, 25].

As for the second approach, a comprehensive review of the existing methods is necessary, if a modification or an introduction of a new method is to be attempted. Mild sonication produced much larger GO than the conventional sonication exfoliation, but the GO was still smaller in size than the pristine graphite [37–39], which suggests that a high degree of fragmentation occurred. On the other hand, gentle shaking [30, 36, 40] generated GO with a size similar [30] to or slightly smaller [40] than the size of the pristine graphite, demonstrating little fragmentation, but the yield was very low. In addition, Ang and co-workers [35] employed refluxing of the mildly oxidized GTO to produce GO, generating a size of 330 μm^2^ (~ 18 μm), but the size of the pristine graphite was not reported, making it difficult to determine whether fragmentation occurred or not. Refluxing was also attempted with large graphite (80 mesh, 178 μm max) in DMF with urea, generating graphene of 10 μm in very low yield [31].

As seen from these, it may not be possible to obtain large-size GO with high yield when the existing methods are employed, even with the use of a large graphite, suggesting that finding a new method may be the better approach. In light of this, shear exfoliation, one of the physical exfoliation methods, received our attention since it is expected to give little or no fragmentation, thereby providing large FLGO. Contrary to expectations, however, small FLGO was reported, suggesting that a high degree of fragmentation may have occurred due to the high-speed blender [10, 19] used for shear exfoliation. It is believed that the blade of the blender exerted high impact force, rather than true shear force, on the graphite oxide (GTO), which resulted in a high degree of fragmentation along with exfoliation.

This led us to seek a new device capable of generating true shear force for exfoliation so that the shear force is parallel to the graphene layer. In this sense, two parallel plates, which move or rotate against each other, appear to be a promising configuration, suggesting a millstone-like device in which the runner stone rotates against the stationary bed stone. In this study, therefore, a new millstone-based device was introduced for the exfoliation of GTO, in an effort to minimize fragmentation and produce large-size FLGO. In addition, mildly oxidized graphite (MOG) was employed to afford FLGO with both good property and high yield, as reported previously [41].

## Methods

### Materials

Natural graphite (325 mesh, 99.8%, metal basis) was purchased from Alfa Aesar (Ward Hill, MA, USA), and KMnO_4_ (ACS reagent, > 99%) was supplied by Sigma-Aldrich (St. Louis, MO, USA). HCl (extra pure, > 35%), H_2_SO_4_ (extra pure, > 95%), and H_2_O_2_ (extra pure, > 35%) were purchased from OCI (Korea).

### Design and Fabrication of Millstone

**Fig. 1:**
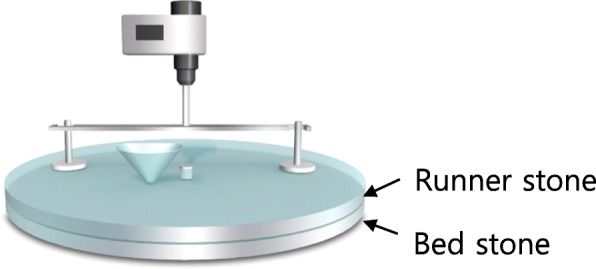
Schematic drawing of millstone for exfoliation of graphite

The millstone (MS) device was designed so that the top glass plate (runner stone) rotates against the stationary bottom plate (bed stone) to generate shear force (Fig. 1). Initially, real stone plates were tested, followed by steel plates, but these were not flat enough to ensure smooth rotation, as well as being difficult to machine into a desired shape. On the other hand, glass plates were much easier for machining, while their transparency made it possible to monitor the progress of exfoliation. A glass plate of 10-mm thickness was cut into a diameter of 35 cm and sandblasted, followed by grinding with an abrasive to ensure a flat and smooth surface. An electric motor with speed controller was attached to the top of the plate to supply power for the rotation.

### Mild Oxidation of Natural Graphite

Mildly oxidized natural graphite (MOG) was prepared via the modified Hummers method, as published earlier [41], and used for MS exfoliation to afford few-layer graphene oxide (FLGO) with good property and high yield. A brief description of the preparation of MOG is as follows: After charging 46 ml of H_2_SO_4_ into a round bottom flask in a 20 °C water bath, 2 g of natural graphite was added, followed by the slow addition of 4 g of KMnO_4_. The mixture was stirred for 30, 60, or 90 min (MOG-30, MOG-60, and MOG-90, respectively) and poured into a 2-l bottle filled with 1.8 l of deionized water (DIW), to which the H_2_O_2_ solution was added.

The MOG solution was transferred to a 2-l PP mesh mess cylinder and the DIW was added to make 2 l. After aging overnight, the top water was decanted and the cylinder was filled with 10% HCl solution. The process of decanting and filling with HCl solution was repeated twice. The process was then repeated three more times with DIW, and the solution was adjusted to have a concentration of 2 mg/ml. Next, the grafting of aryl diazonium salts of sulfonic acid (ADS) was carried out via the one-step process to enhance the water dispersion, as reported earlier [42]. Finally, the solution was cleaned by centrifugation at 4 k rpm for 30 min to remove the unreacted ADS.

### Exfoliation of MOG via Millstone

MS exfoliation was carried out first on the MOG-60 solution by varying the rpm and the concentration of MOG solution to maximize the yield of FLGO with minimum fragmentation. First, rpm of the top plate was changed from 10 to 50 with 10 ml of 1 mg/ml solution. Approximately 1 ml of the aqueous MOG solution was fed into the funnel attached to the top plate, followed by rotation at given rpm. When the solution was consumed, another 1 ml was added to the funnel, and the process was repeated until all of the 10-ml solution was consumed. At the end of exfoliation, 10 ml of the DIW was used to wash out the residual FLGO that may remain between the two glass plates.

Next, the effect of solution concentration (0.5, 1, and 2 mg/ml) was studied at 30 rpm, as described above. In addition, further exfoliation was also attempted by repeating the MS exfoliation two or three times with the same solution. A solution of 1 mg/ml concentration was used at 30 rpm, and washing with DIW was carried out only once after the final exfoliation. Reproducibility was confirmed by carrying out MS exfoliation at least three times for each solution. Finally, the solutions of MOG-30 and MOG-90 were also studied for comparison. The solutions were subjected to dialysis using a cellulose membrane (Spectrum Labs, *d* = 25.5 mm, MWCO = 6–8 kDa) to prepare the samples for TEM and AFM analysis and for sheet resistance measurements.

### Characterization of MOG and FLGO

UV-vis spectroscopy (Agilent, 8453) was carried out for measuring the absorption at 660 nm for MOG solutions after oxidation and after exfoliation. TEM (Jeol-2100, Japan) and AFM (XE-100, Park Systems, Korea) were also utilized for the characterization of FLGO. Field emission scanning electron microscopy (FE-SEM, Jeol, JSM-7500F, Japan) was carried out at 10 keV for the characterization of MOG and exfoliated MOG by using samples coated with Pt. The sheet resistance was measured by the 4-point probe method (CMT-SR2000, Changmin Tech., Korea), and the sample weight was controlled to ~ 2 mg. At least three samples were measured and the results were averaged. The yield was calculated from the weight of the precipitate and supernatant after the centrifugation of the 10-ml solution at 1 k rpm for 2 h. In addition, the yield was also calculated from the UV-vis absorption of the top solution after centrifugation and of the MOG solution before centrifugation.

## Results and Discussion

### Mild Oxidation of Natural Graphite

**Fig. 2:**
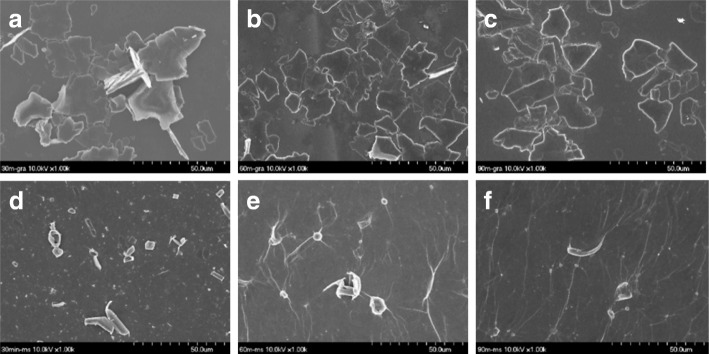
SEM micrographs of mildly oxidized graphite and MS-exfoliated MOG, **a** MOG-30, **b** MOG-60, **c** MOG-90, **d** MOG-30-MS, **e** MOG-60-MS, and **f** MOG-90-MS

Mildly oxidized graphite (MOG) solutions prepared by the modified Hummers method provided UV-vis absorptions of 7.8, 9.9, and 11.2 for MOG-30, MOG-60, and MOG-90, respectively (these are slightly higher than the values from the previous study [41] since they were measured at 660 nm rather than at 750 nm), indicating mild oxidation. As expected from the previous study [41], there was a high degree of oxidation-exfoliation, but fairly thick MOG plates with some edge-expanded MOG were observed from all samples, demonstrating that mild oxidation took place (Additional file 1: Figure S1). SEM micrographs after ADS grafting showed fairly thin (~ 1 μm) MOG plates (Fig. 2) for all samples, but only MOG-30 showed the edge-expanded structure (Fig. 2a), indicating that further exfoliation took place during the grafting process. The SEM analysis also revealed a lateral size of MOG as large as ~ 20 μm, which can be compared with the size of large (20~30 μm) and small (< 10 μm) graphite particles of the as-received sample (Additional file 1: Figure S1d). This shows that little fragmentation occurred upon oxidation, possibly due to mild oxidation.

### Optimization of Rotation Speed for MS Exfoliation

**Fig. 3:**
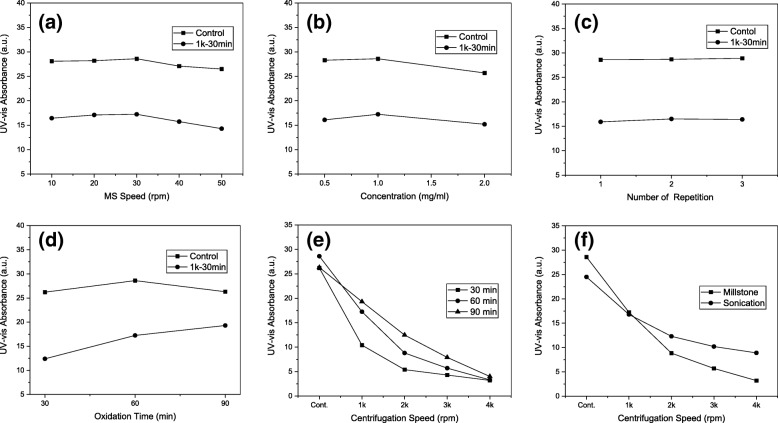
UV-vis absorbance of MS-exfoliated MOG solution. Effect of **a** MS rotation speed, **b** solution concentration, **c** number of repetition, **d** oxidation time, and **e** oxidation time with centrifugation rpm and **f** comparison of MS and sonication exfoliation

First, the rpm of MS exfoliation was optimized by varying it from 10 to 20, 30, 40, and 50 using the MOG-60 solution of 1 mg/ml concentration. As the rpm increased, the absorption remained almost unchanged at 10, 20, and 30 rpm and then decreased slightly at 40 and 50 rpm, providing UV-vis absorption of 28.1, 28.2, 28.6, 27.2, and 26.5, respectively (Fig. 3a). A similar trend was also observed from the solution after centrifugation at 1 k rpm for 30 min (Fig. 3a). It is noted that this is very similar to the Stribeck curve which shows an unchanged friction coefficient at the boundary regime but a decreased friction coefficient in the mixed regime as the speed increases. Therefore, one can say that the UV-vis absorption at 10, 20, and 30 rpm remains virtually unchanged because of the nearly constant friction force in the boundary regime, while the UV-vis absorption at 40 and 50 rpm is decreased due to the decreased friction force in the mixed regime, leading to the decreased exfoliation of MOG plates.

Moreover, the time required for MS exfoliation at 10 rpm was 90 min, while 70, 45, 35, and 25 min were needed at 20, 30, 40, and 50 rpm, respectively. Based on such considerations, the rpm of 30 was selected as the optimum rpm for this study. As noted, the UV-vis absorption of MS-exfoliated samples exhibited as high as ~ 300% increase (for 30 rpm), demonstrating the excellent exfoliation capability of the proposed MS.

### Optimization of MOG Solution Concentration

When the concentration of MOG solution was varied from 1 mg/ml to 0.5 or 2 mg/ml at 30 rpm, the UV-vis absorption of 28.3 and 27.7 was obtained, respectively (Fig. 3b). It is believed that the former value is similar to the absorption of 28.6 obtained from the 1 mg/ml solution, since the concentration is low enough (0.5 mg/ml) to allow the complete separation of each MOG plate without stacking. This would provide maximum shear force for each MOG plate and lead to a maximum degree of exfoliation. On the other hand, the slightly lower absorption from the 2 mg/ml solution can be attributed to the presence of some stacking in the MOG plates due to the high concentration, which resulted in sliding of the MOG plates and thus deceased degree of exfoliation. Despite the similar results obtained from 0.5 and 1 mg/ml solutions, the latter was preferred since much shorter time was needed for exfoliation (45 min as opposed to 80 min for the former). The UV-vis absorption after centrifugation at 1 k rpm for 30 min also exhibited the same trend, supporting the selection of 1 g/ml solution as the optimum concentration for this study.

### Effect of Repetition of MS Exfoliation

Since MS exfoliation was carried out only once to optimize the aforementioned conditions, an attempt was made to determine if repeated exfoliations would lead to further exfoliations. When the MS exfoliation was carried out on the MOG-60 solution of 1 mg/ml concentration at 30 rpm, the UV-vis absorption of 28.3, 28.6, and 28.9 was obtained for one, two, and three rounds of exfoliation, respectively (Fig. 3c). It can be seen that the values are very similar to each other, suggesting that repeated exfoliations are not necessary. The absorption after centrifugation at 1 k rpm for 30 min exhibited a similar trend. Therefore, a single round of exfoliation was selected, along with 1 mg/ml concentration and 30 rpm, as the optimum conditions.

### Effect of Oxidation Time on MS Exfoliation

The solutions of MOG-30 and MOG-90 under the optimum condition showed UV-vis absorptions of 26.2 and 26.3, respectively, which are slightly lower than 28.6 from MOG-60 (Fig. 3d). The lower absorption with MOG-30 can be attributed to the lower degree of exfoliation, possibly due to the lower degree of oxidation. However, the lower absorption with MOG-90 can be explained by the damaged sp^2^ carbon bonds resulting from a high degree of oxidation, despite the higher degree of exfoliation, since the damaged sp^2^ carbon bond does not contribute to the UV-vis absorption. This trend is similar to what was reported for the sonication exfoliation of MOG solution [41].

It is interesting to note that the UV-vis absorption increased with oxidation time after centrifugation (Fig. 3d), indicating that the number of FLGO increased due to the increased exfoliation with oxidation time. This is different from what was observed before centrifugation and can be explained by the degree of exfoliation that increases in the order of MOG-30, MOG-60, and MOG-90, which in turn resulted in the highest number of FLGO with MOG-90, followed by MOG-60 and MOG-30.

As the centrifugation rpm increased, the UV-vis absorption of MOG-90 decreased almost linearly (Fig. 3e), suggesting a nearly uniform distribution of FLGO (size or weight). On the other hand, MOG-60 showed a rather rapid decrease at low rpm but relatively slow decrease at high rpm. A similar behavior was observed from MOG-30 but with a much faster decrease at low rpm. This can be explained by the number of heavy (or large) FLGO present, which decreases in the order of MOG-30, MOG-60, and MOG-90 solution, and indicated that a lower degree of oxidation resulted in a lower degree of exfoliation. As noted, however, very similar UV-vis absorptions were observed at 4 k rpm centrifugation, regardless of the oxidation time, suggesting that a very low degree of fragmentation occurred upon MS exfoliation for all solutions.

On the other hand, the UV-vis absorption of the MS-exfoliated MOG-60 solution showed a very different behavior (Fig. 3f) than the one from the same solution after sonication exfoliation (24 h). The latter showed a much slower decrease in the UV-vis absorption, which is attributed to a much higher number of small FLGO resulting from the higher degree of fragmentation via sonication. This was supported by the small size (< 1 μm) of FLGO after sonication, as previously reported [43].

**Fig. 4:**
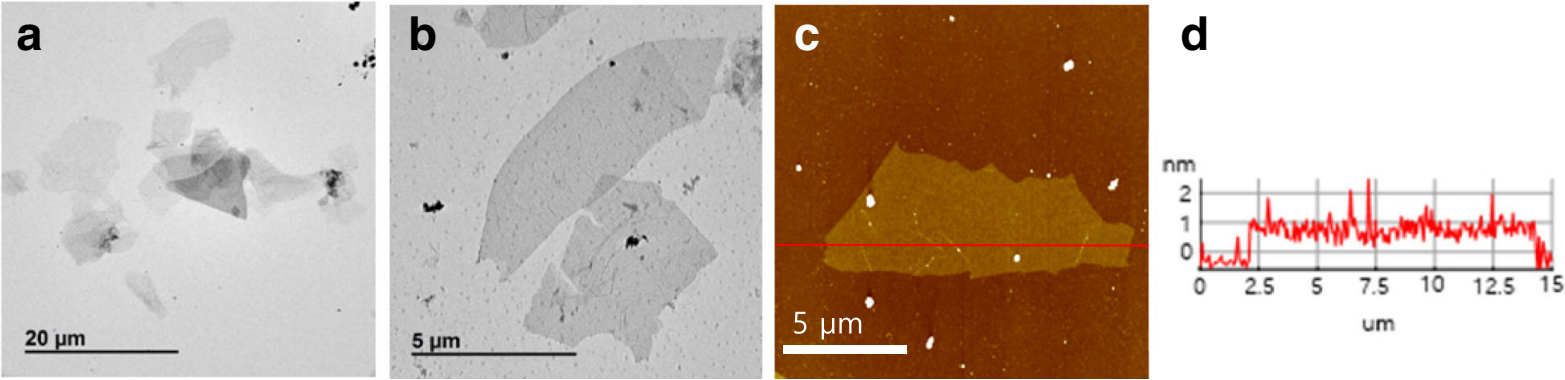
TEM (**a, b**) and AFM micrograph (**c**) of MS-exfoliated MOG-60 and thickness profile (**d**)

The SEM analysis revealed thin MOG plates (before exfoliation), which were no longer observed after MS exfoliation (Fig. 2). However, rolled or partially rolled MOG plates were observed occasionally in all MOG solutions (Fig. 2). Such structures are believed to be formed via shear force from MS exfoliation. On the other hand, the TEM analysis of FLGO from MOG-60 showed a lateral size as large as ~ 10 μm (Fig. 4a, b), and similar TEM micrographs were obtained from MOG-30 and MOG-90 (Additional file 1: Figure S2). Considering the size of graphite (20–30 μm) (Additional file 1: Figure S1d), there may be some fragmentation upon MS exfoliation. This can be compared with sonication exfoliation, which showed ~ 1 μm or smaller size FLGO, in general [43]. Of course, many smaller FLGOs (< 10 μm) are also present after MS exfoliation, likely due to the small size of the as-received graphite, along with some degree of fragmentation. The AFM analysis also showed FLGO (MOG-60) with a similar size (~ 10 μm) as that observed in the TEM, demonstrating successful exfoliation with little fragmentation (Fig. 4c). In addition, the AFM revealed a thickness of ~ 1 nm, which corresponds to ~ 3 layers, indicating good exfoliation. Similar AFM results were also obtained from MOG-30 and MOG-90, as expected (Additional file 1: Figure S3). Of course, much thicker FLGO was also observed in both the TEM and the AFM.

The calculated yield of FLGO increased with oxidation time, providing 36, 51, and 65% for MOG-30, MOG-60, and MOG-90, respectively. These values can be compared with 35, 47, and 56% obtained from UV-vis absorption measurements. It can be seen that the yield based on weight is higher than the yield from UV-vis absorption. This is attributed to FLGO with damaged sp^2^ carbon bonds, which can contribute to the weight but not to the UV-vis absorption. When compared with 19, 55, and 73% yield obtained from sonication exfoliation [41], one can see that the yield is higher, similar, or lower for MOG-30, MOG-60, or MOG-90, respectively. The higher yield with MOG-30 can be attributed to true shear exfoliation, which is much less affected by the degree of oxidation than sonication exfoliation. In other words, sonication exfoliation is strongly dependent on the degree of oxidation due to its fragmentation-induced exfoliation. Lastly, the sheet resistance measured was 3.2 × 10^2^, 4.3 × 10^3^, and 2.5 × 10^4^ Ω/□ for MOG-30, MOG-60, and MOG-90, respectively (Table 1). As expected, these values increased with oxidation time and were similar to the values obtained from sonication exfoliation. Such results may be explained by the presence of only a few large FLGO, which did not have a significant impact on the sheet resistance.

**Table 1:** Sheet resistance and yield of FLGO from MS exfoliation

	MOG-30	MOG-60	MOG-90
Rs (Ω/□)	3.2 ± 0.8 × 10^2^	4.3 ± 0.6 × 10^3^	2.5 ± 1.5 × 10^4^
Yield (%)^a^	36	51	65
Yield (%)^b^	35	47	56

^a^Based on weight from top solution and bottom precipitate upon centrifugation

^b^Based on UV-vis absorption from top solution after centrifugation and the solution before centrifugation

## Conclusions

A millstone was successfully fabricated with two glass plates and utilized for the exfoliation of mildly oxidized graphite. The optimum conditions for MS exfoliation, which were obtained by varying the rotational rpm (10–50), solution concentration (0.5–2 mg/ml), and number of exfoliation (1–3 times), were 30 rpm, 1 mg/ml, and one round of exfoliation. The TEM and AFM analysis showed very thin FLGO (~ 1 nm) with a size of ~ 10 μm and indicated that successful exfoliation took place with little fragmentation, compared with the pristine graphite (20–30 μm). The SEM analysis revealed edge-rolled FLGO occasionally, which was attributed to true shear exfoliation. The yield of FLGO from the weight measurement was 36, 51, and 65% for MOG-30, MOG-60, and MOG-90, respectively. A comparison with 19, 55, and 73% obtained from sonication exfoliation showed that a much better exfoliation occurred for MOG-30 with MS exfoliation, likely due to true shear exfoliation. The sheet resistance, however, was similar to the previously reported results and indicated that the number of large FLGO obtained via MS was not large.

## Additional file

Additional file 1: **Figure S1.** SEM micrographs from MOG-30, MOG-60, MOG-90, and as-received graphite. **Figure S2.** TEM micrographs from MOG-30 and MOG-90. **Figure S3.** AFM micrographs from MOG-30 and MOG-90 (DOCX 2127 kb)
