# Supplementary material for: Millstone Exfoliation: a True Shear Exfoliation for Large-Size Few-Layer Graphene Oxide
Source: Nanoscale Res Lett. 2018 Jun 20;13:186. doi: 10.1186/s11671-018-2598-y (PMC6010364; doi:10.1186/s11671-018-2598-y)
Supplement: Supplementary file 1 — Figure S1. SEM micrographs from MOG-30, MOG-60, MOG-90, and as-received graphite. Figure S2. TEM micrographs from MOG-30 and MOG-90. Figure S3. AFM micrographs from MOG-30 and MOG-90 (DOCX 2127 kb) [file 11671_2018_2598_MOESM1_ESM.docx]

**Supporting Information**

**Millstone exfoliation: a true shear exfoliation for large size few-layer-graphene oxide**

Heng-Ju Yoon, Jae Young Lee and Tae-Ho Yoon***

School of Materials Science and Engineering, Gwangju Institute of Science and Engineering (GIST), 123 Cheomdangwagi-ro, Buk-gu, Gwangju, 61005, South Korea.

* To whom correspondence should be addressed (thyoon@gist.ac.kr, Fax: +82-62-715-2324, Tel:+82-62-715-2307)


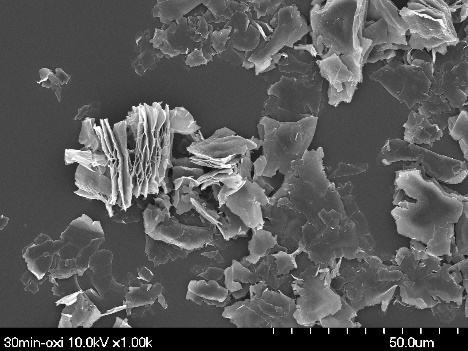

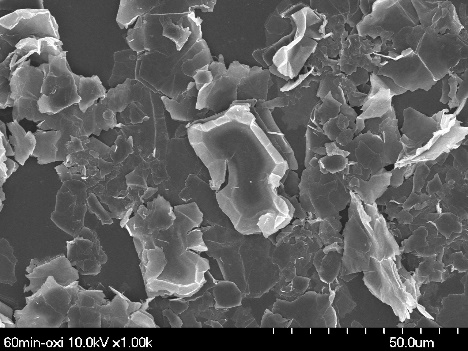

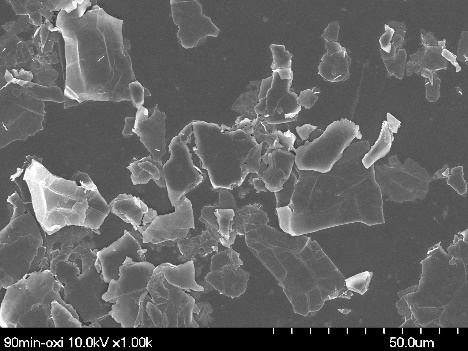

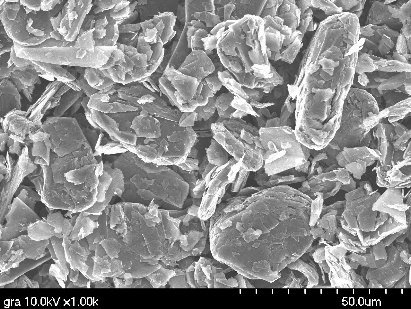


**(b)**

**(c)**

**(d)**

**(a)**

Fig. S1. SEM micrographs from MOG-30 (a), MOG-60 (b), MOG-90 (c) and as-received graphite (d).


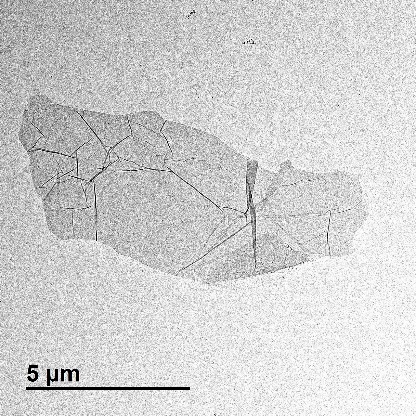

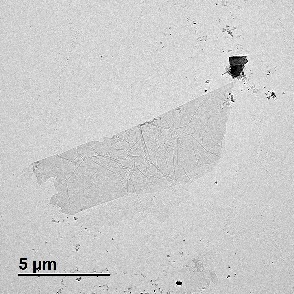


**(a)**

**(b)**

Fig. S2.TEM micrographs from MOG-30 (a) and MOG-90(b).


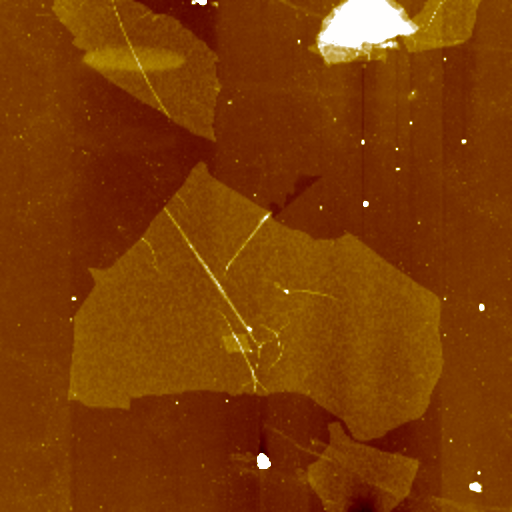

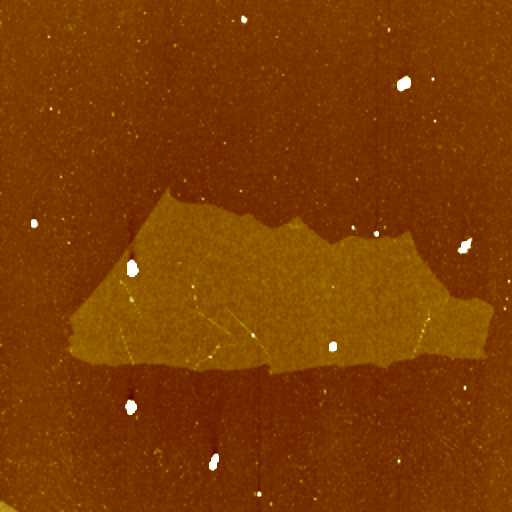


**(a)**

**(b)**

**5 µm**

**5 µm**


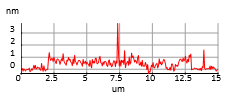

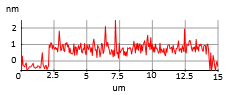


Fig. S3. AFM micrographs from MOG-30(a) and MOG-90(b).
